# Better Understanding the Timing Of Androgen Deprivation Trial Outcomes: Impacts of Prior Androgen Deprivation Therapy

**DOI:** 10.1093/jncics/pkac025

**Published:** 2022-03-28

**Authors:** Kristian D Stensland, Theresa Devasia, Megan E V Caram, Christina Chapman, Alexander Zaslavsky, Todd M Morgan, Brent K Hollenbeck, Jordan B Sparks, Jennifer Burns, Varsha Vedapudi, Gillian M Duchesne, Alexander Tsodikov, Ted A Skolarus

**Affiliations:** 1 Dow Division of Health Services Research, Department of Urology, University of Michigan, Ann Arbor, MI, USA; 2 Department of Urology, Division of Urologic Oncology, University of Michigan, Ann Arbor, MI, USA; 3 Department of Biostatistics, School of Public Health, University of Michigan, Ann Arbor, MI, USA; 4 Department of Medicine, Division of Oncology, University of Michigan, Ann Arbor, MI, USA; 5 Health Services Research & Development Center for Clinical Management Research, VA Ann Arbor Healthcare System, Ann Arbor, MI, USA; 6 Department of Radiation Oncology, University of Michigan, Ann Arbor, MI, USA; 7 Sir Peter MacCallum Department of Oncology, University of Melbourne, Melbourne, Victoria, Australia; 8 Rogel Cancer Center, University of Michigan, Ann Arbor, MI, USA

## Abstract

**Background:**

The Timing Of Androgen Deprivation (TOAD) trial found an overall survival benefit for immediate vs delayed androgen deprivation therapy (ADT) for prostate-specific antigen (PSA)–relapsed or noncurable prostate cancer. However, broad eligibility criteria allowed entry of a heterogeneous participant group, including those with prior ADT exposure, raising concerns about subsequent androgen sensitivity. For these reasons, we completed previously specified subgroup analyses to assess if prior ADT was associated with ADT timing efficacy after PSA relapse.

**Methods:**

We examined TOAD trial patient-level data for participants with PSA relapse after local therapy. We performed Kaplan-Meier analyses for overall survival stratified by prior ADT and randomized treatment arm (immediate or delayed ADT). We compared group characteristics using Mann-Whitney U and Fisher exact tests. All hypothesis tests were 2-sided.

**Results:**

We identified 261 patients with PSA relapse, 125 of whom received prior ADT. Patients with prior ADT had higher PSA at presentation (12.1 vs 9.0 ng/mL; *P* < .001), more cT3 disease (38.4% vs 25.0%; *P* = .007), and more likely received radiotherapy as local treatment (80.0% vs 47.8%; *P* < .001) but were otherwise similar to patients without prior ADT exposure. Within this prior ADT group, those who received immediate ADT (n = 56) had improved overall survival compared with those who received delayed ADT (n = 69; *P* = .02). This benefit was not observed in the group with no prior ADT (*P* = .98).

**Conclusions:**

The survival benefit demonstrated in the TOAD trial may be driven by patients who received ADT prior to trial entry. We provide possible explanations for this finding with implications for treatment of PSA-relapsed prostate cancer and future study planning.

Androgen deprivation therapy (ADT) is a mainstay of treatment for men with prostate-specific antigen (PSA) relapse after curative therapy (1,2). However, when to start ADT after PSA relapse remains unclear. For example, the 2021 National Comprehensive Cancer Network Prostate Cancer guidelines state: “Earlier ADT may be better than delayed ADT. . . . Since the benefit of early ADT is not clear, treatment should be individualized until definitive studies are done” (1).

The Timing Of Androgen Deprivation trial (ie, TOAD) was a randomized clinical trial seeking to address this issue by comparing the effect of immediate vs delayed ADT on overall survival, primarily among men with PSA relapse (3). The TOAD trial found an overall survival benefit associated with immediate ADT. Among other criticisms, a remaining limitation is the possibility of differential benefit within patient subgroups (4,5). This was addressed, in part, by stratified randomization according to initial treatment (surgery vs radiotherapy), time to relapse, and PSA doubling time. Another important biological consideration rests on prior ADT receipt and subsequent ADT response (ie, potentiation) (6). In other words, whether prior ADT exposure was associated with differential survival effects remains unclear, though relevant to ADT timing and castration resistance. In fact, nearly half of study participants with PSA relapse received prior ADT and, because of randomization, were balanced across the early and delayed ADT arms creating an opportunity to examine the underlying hypothesis that prior ADT may alter subsequent biological response in favor of immediate use. Although prior receipt of ADT was prespecified for subanalysis as a prognostic factor in the TOAD trial protocol, to date these results have not been reported (7).

To better understand this issue and inform definitive studies of timing of ADT for PSA relapse, we completed a prespecified subanalysis of the TOAD trial data. We explored how survival outcomes varied according to prior ADT exposure across immediate and delayed groups and suggest biological and behavioral hypotheses and potential implications for the timing and use of ADT among men with PSA relapse.

## Methods

### Description of the TOAD Trial

The TOAD trial recruited 293 men between September 2004 and July 2012. Eligible patients were post local therapy with PSA relapse (n = 261) or had disease unsuitable for local treatment (n = 32). Patients were randomly assigned to immediate ADT or delayed ADT with a recommended interval of 2 years prior to ADT initiation. PSA relapse was defined per guidelines depending on prior treatment—radiotherapy, surgery, and/or ADT in any combination. Patients who received prior ADT were eligible for inclusion if it was delivered more than 1 year prior to study entry and the duration of prior ADT was less than 1 year. Of note, this means high-risk patients receiving currently recommended 18-24 months of periradiation ADT are either not reflected in the trial or received less than currently recommended ADT dosing during their local treatment. Randomization was stratified on prior treatment (surgery vs radiotherapy), time to relapse (less than vs greater than 2 years), and PSA doubling time (less than vs greater than 10 months) but not on prior ADT exposure. However, prior ADT exposure was prespecified as a prognostic factor of interest for further inspection of possible interactions with the effect of the timing of ADT delivery on survival (7).

Overall survival for the study population was 91% in the immediate ADT group compared with 86% in the delayed ADT group (*P* = .05). Importantly, the study did not show a statistically significant survival benefit for immediate ADT among the PSA-relapse group (*P* = .10). This latter group represented most study participants and was the focus of our investigation.

### Statistical Methods

We analyzed individual patient data from the TOAD trial (3). We limited our analyses to the PSA-relapse cohort, excluding those without prior local treatment. We compared prior ADT and no prior ADT groups with respect to demographics, including Gleason score and tumor, node, and metastasis scores at diagnosis; PSA doubling time; PSA at time of diagnosis and trial entry; and time to ADT using Fisher exact test for categorical variables and Mann-Whitney U test for continuous variables. We performed Kaplan-Meier analyses for overall survival stratified by prior ADT and randomized treatment arm (immediate or delayed ADT). We estimated prostate cancer–specific mortality with other-cause mortality as a competing risk using the cumulative incidence function. We then estimated prostate cancer–specific survival using a competing risks regression with other-cause mortality as a competing risk and treatment arm, prior ADT, and age at randomization as covariates with a treatment arm * prior ADT interaction term.

We tested for heterogeneity of treatment effects to assess for subgroup benefit through a Cox proportional hazards model, adjusting for age and planned ADT schedule as described in the TOAD trial analysis (see Table 2) and assessing the interaction term between prior ADT and randomized group (ie, immediate vs delayed ADT) (8). Although this interaction term was not statistically significant (*P* = .09), we continued our exploratory analysis of this subgroup given the large effect estimate (hazard ratio [HR] = 0.29), causal rationale for heterogeneity, and the prespecified nature of the prior ADT subgroup analysis. Finally, we conducted sensitivity analyses according to initial treatment (surgery, radiation) to inform our biological hypotheses.

**Table 2.** Cox proportional hazards model for overall survival within PSA-relapse group

Covariate	Hazard ratio (95% CI)	*P* ^a^
Immediate treatment	1.00 (0.41 to 2.40)	.99
Prior ADT	2.00 (0.90 to 4.44)	.09
Immediate treatment * prior ADT	0.29 (0.07 to 1.2)	.09
Age at randomization	0.99 (0.95 to 1.04)	.68
Planned intermittent ADT	0.86 (0.45 to 1.65)	.66

a Hypothesis tests computed via 2-sided Wald test. ADT = androgen deprivation therapy; PSA = prostate-specific antigen.

All analyses were conducted using R version 4.0.3 (R Core Team, Vienna, Austria). Statistical significance was considered at alpha equal to 0.05; all hypothesis tests were 2-sided. This study was approved by the VA Ann Arbor Healthcare System and University of Michigan institutional review boards.

## Results

As shown in Table 1, participants who received prior ADT had a higher proportion of radiotherapy as definitive treatment (80.0% vs 47.8%; *P* < .001) and had more aggressive prostate cancer features at time of local treatment than the no prior ADT group, including higher PSA levels at diagnosis (median 12.1 vs 9.0 ng/mL; *P* < .001) and higher percentage of T3 disease (38.4% vs 25.0%; *P* = .007). However, characteristics at time of trial entry were similar between groups including PSA (*P* = .13), distribution of Gleason scores (*P* = .09), PSA doubling time (*P* = .30), and median follow-up of approximately 5 years (*P* = .08).

**Table 1.** Characteristics of PSA-relapsed TOAD cohort, stratified by receipt of prior ADT

Variable	No prior ADT (n = 136)	Prior ADT (n = 125)	*P* ^a^
Age at randomization, median (IQR), y	70.5 (64.8-75.1)	72.1 (66.3-77.3)	.08
Immediate ADT, No. (%)	68 (50.0)	56 (44.8)	.47
T stage, No. (%)			.007
T1	35 (25.7)	18 (14.4)	
T2	63 (46.3)	58 (46.4)	
T3	34 (25.0)	48 (38.4)	
T4	0 (0)	1 (0.8)	
Tx	4 (2.9)	0 (0)	
Follow-up median (IQR), mo	5.0 (3.5-6.3)	4.6 (3.0-6.1)	.08
PSA at time of trial entry, median (IQR)	2.8 (0.6-4.5)	3.2 (1.1-4.9)	.13
PSA at presentation, median (IQR)	9.0 (6.6-12.7)	12.1 (7.5-19.7)	<.001
PSA doubling time; median (IQR), mo	11.1 (6.1-18.0)	10.6 (6.3-14.2)	.30
Gleason score, No. (%)			.09
<6	13 (9.7)	5 (4.0)	
6	28 (20.9)	19 (15.2)	
7	69 (51.5)	67 (53.6)	
8	9 (6.7)	18 (14.4)	
9	15 (11.2)	16 (12.8)	
Definitive treatment, No. (%)			<.001
Radiotherapy	65 (47.8)	100 (80.0)	
Surgery	11 (8.0)	1 (0.8)	
Surgery + radiotherapy	60 (44.1)	24 (19.2)	

a Hypothesis tests for continuous variables (age, follow-up, PSA at time of trial entry and presentation, PSA doubling time) computed with 2-sided Kruskal-Wallis test. Hypothesis tests for categorical variables (immediate ADT, T stage, Gleason score, definitive treatment type) computed with 2-sided Fisher exact test. ADT = androgen deprivation therapy IQR = interquartile range; PSA = prostate-specific antigen; TOAD = Timing Of Androgen Deprivation.

There were 40 deaths from any cause and 18 deaths from prostate cancer (PCa) in the study period. These included 6 PCa and 4 other-cause deaths in the no prior ADT-delayed ADT group, 5 PCa and 5 other-cause deaths in the no prior ADT-immediate ADT group, 6 PCa and 10 other-cause deaths in the prior ADT-delayed ADT group, and 1 PCa and 3 other-cause deaths in the prior ADT-immediate ADT group.

**Figure 1.**
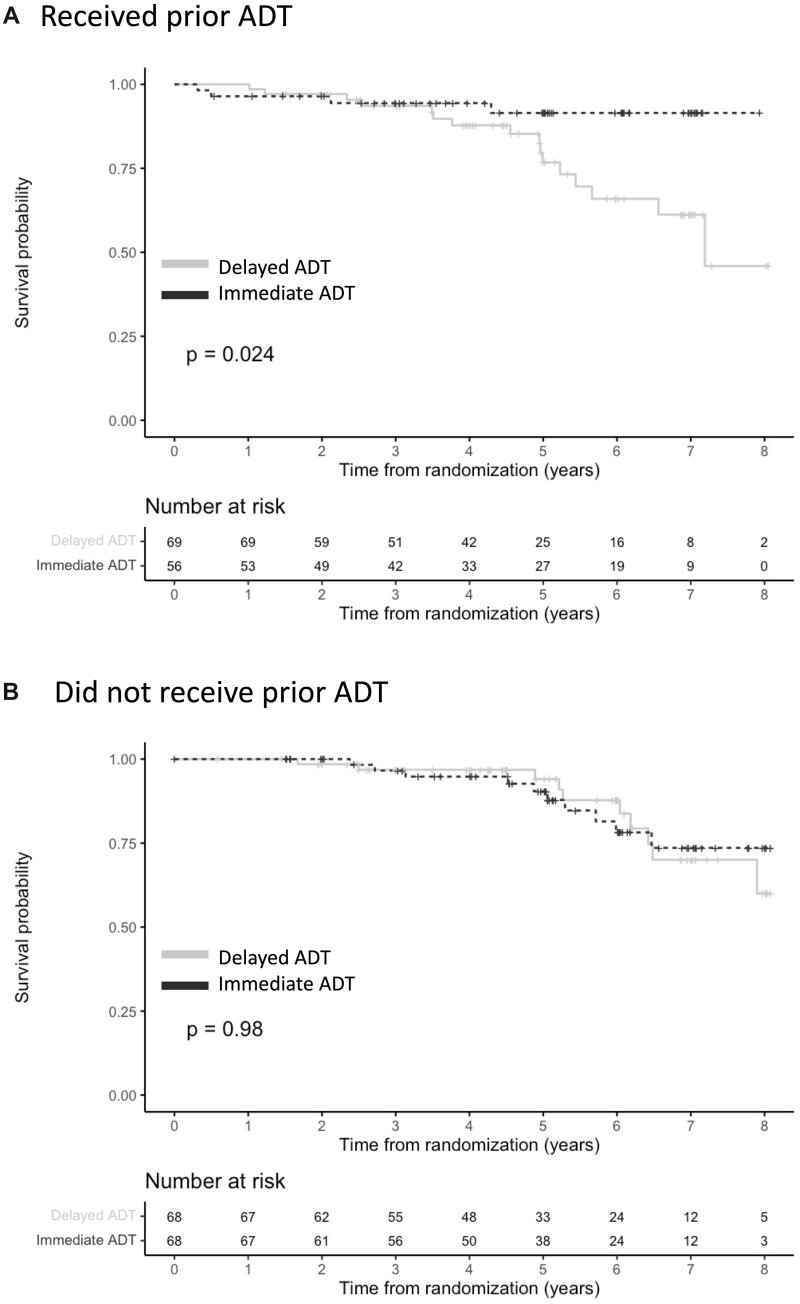
Overall survival for immediate vs delayed ADT, stratified by receipt of prior ADT. **A)** Overall survival for immediate vs delayed ADT at time of recurrence for patients with prior ADT. **B)** Overall survival for immediate vs delayed ADT at time of recurrence for patients with no prior ADT. ADT = androgen deprivation therapy.

**Figure 2.**
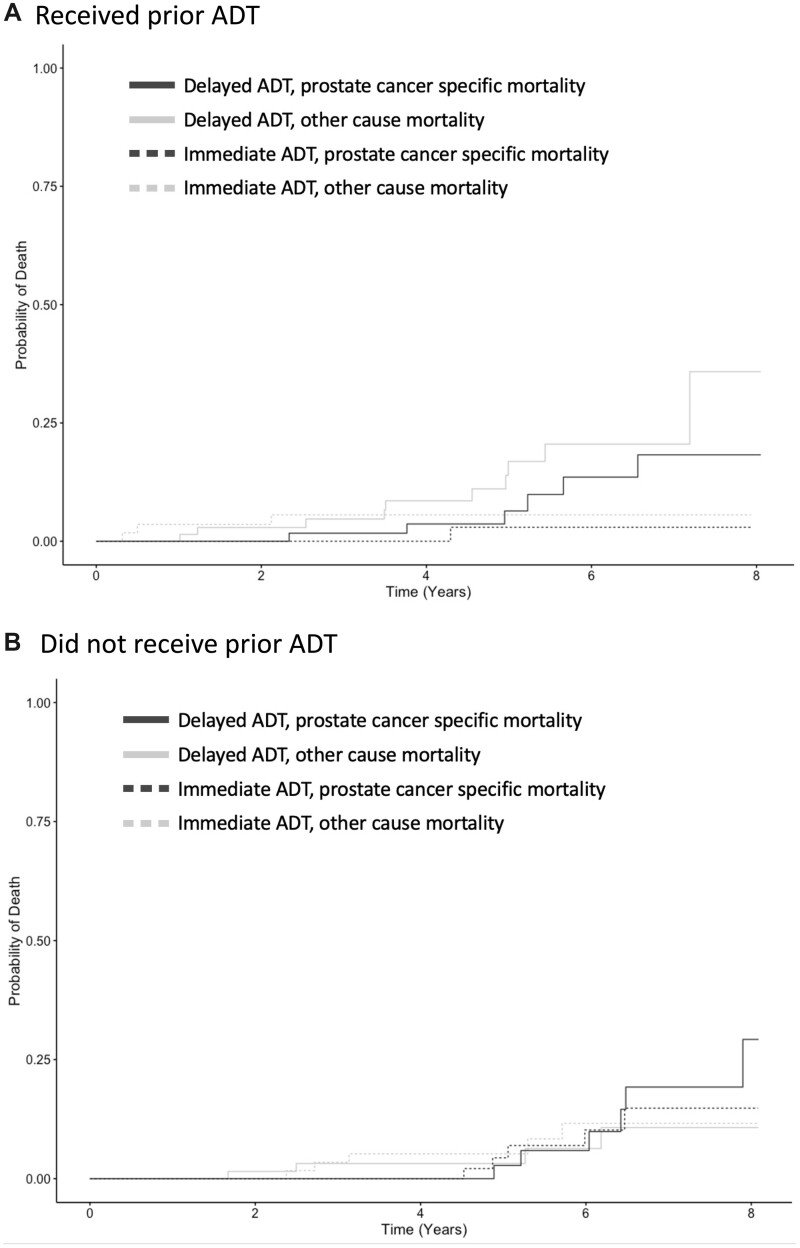
Prostate cancer–specific mortality for immediate vs delayed ADT, stratified by receipt of prior ADT. **A)** Prostate cancer–specific mortality for immediate vs delayed ADT at time of recurrence for patients with prior ADT. **B)** Prostate cancer–specific mortality for immediate vs delayed ADT at time of recurrence for patients with no prior ADT. ADT = androgen deprivation therapy.

Among participants who received prior ADT, the group randomized to immediate ADT at time of relapse had better overall survival compared with the delayed ADT group (*P* = .02; Figure 1, A). In contrast, in patients without prior ADT, overall survival for immediate vs delayed ADT was similar (*P* = .98; Figure 1, B). Prostate cancer–specific survival appeared longer for immediate compared with delayed ADT in patients with prior ADT but was not statistically significant (*P* = .11; Figure 2, A) and was indistinguishable in those without prior ADT (*P* = .73; Figure 2, B).

On multivariable analysis (Table 2), we found a large but non-statistically significant interaction between the timing of ADT (immediate vs delayed) and receipt of prior ADT (adjusted hazard ratio [HR] = 0.29, 95% confidence interval [CI] = 0.07 to 1.2; *P* = .09). This suggests patients who received prior ADT then immediate ADT at time of relapse may have a lower hazard of death. On competing risk analysis, there was similarly a large but non-statistically significant interaction between timing of ADT and receipt of prior ADT with prostate cancer–specific mortality (HR = 0.26, 95% CI = 0.02 to 2.98; *P* = .28).

In sensitivity analyses to examine the impact of local definitive therapy on immediate vs delayed ADT, we limited our cohort to patients treated definitively with radiotherapy (n = 165). In this subset, there were 2 PCa and 2 other-cause deaths in the no prior ADT-delayed ADT group, 4 PCa and 3 other-cause deaths in the no prior ADT-immediate ADT group, 6 PCa and 9 other-cause deaths in the prior ADT-delayed ADT group, and 1 PCa and 3 other-cause deaths in the prior ADT-immediate ADT group. This suggested benefit for immediate vs delayed ADT only in the prior ADT group as in the overall analysis but was not statistically significant (*P* = .10). In patients treated with surgery or surgery and radiotherapy, we did not see a survival benefit for immediate ADT (*P* = .42). However, within this subgroup, only 26% of participants received prior ADT limiting our power to detect differences.

## Discussion

In this post hoc analysis of the original TOAD trial data, we found immediate ADT was associated with survival benefits only among patients previously exposed to ADT. In other words, we found an interaction between the timing of subsequent ADT (immediate vs delayed) and receipt of prior ADT adding novel insights into the timing and use of ADT for men with PSA relapse after definitive treatment. For men with prior ADT exposure and PSA relapse, we found earlier ADT may be better when it comes to survival outcomes. Conversely, for men with no prior ADT exposure, we did not find any differences as to the timing of ADT, potentially creating opportunities to delay ADT and its harmful side effects until clinical indicators might warrant treatment (eg, PSA doubling time). Through our sensitivity analyses, we were unable to identify obvious confounding factors to explain these important differences, aside from paradoxically more aggressive tumor features at the time of local treatment in the subgroup with the best survival. Although limitations to our post hoc analyses exist, we believe the magnitude of our effect sizes surrounding immediate ADT use according to prior ADT exposure is hypothesis generating with respect to biological and behavioral mechanisms warranting further study.

First, there may be a biological causal pathway for the observed survival difference. Castration with ADT is known to change both prostate cancer cells and the tumor microenvironment, ultimately leading to progression and castration resistance (9-12). It is possible that similar changes could also “prime” cancer cells and androgen receptors to preferentially benefit from immediate ADT at the time of recurrence through potentiation. These evoked differential expressions of androgen receptors and other prostate cell components are used to support studies of new androgen-directed and immune therapy for prostate cancer (13,14). The initial receipt of ADT at time of local therapy may have caused such changes to tumor cells or the surrounding microenvironment, resulting in a recurrent disease phenotype that preferentially responded to immediate ADT. It is also possible that the initial receipt of ADT selected for a specific population of cells that preferentially responded to immediate ADT at recurrence. This is similar to previously described mechanisms of preexisting drug-resistant or prostate cancer stem–like cells described in analyses of progression to castration resistance (6,15). In both of these proposed mechanisms, improved survival in the immediate ADT group was potentiated by prior receipt of ADT, but the cause of the timing effects remains unclear. It is possible that the sensitization by prior ADT results in a brief window of enhanced sensitivity when local cells or undetected micrometastases must be treated with ADT prior to progression out of the window of androgen sensitivity. These hypotheses are speculative and must be assessed in future studies.

Conversely, biological changes caused by ADT may induce harmful mutations leading to aggressive cell transformation and progression. Such changes have been suggested as mechanisms for drug resistance and avoidance of senescence (16). Similarly, modeling studies suggest cancer therapies such as radiation can possibly induce cancer development and progression (17). It is possible that exposure to ADT at the time of local treatment provokes development of cells that are more volatile, leading to enhanced response to immediate ADT, but also more aggressive and lethal if left untreated (ie, delayed ADT). This hypothesis is supported by the group with prior ADT randomized to delayed ADT showing the worst overall survival of any group. This hypothesis would also help explain the similar 5-year survival from prior cohorts with low rates of ADT at time of recurrence, such as Freedland et al. (18) wherein only 14% of postprostatectomy patients with biochemical recurrence received hormonal therapy prior to developing metastatic disease, yet 5-year survival was 93%. However, this hypothesis requires further study to assess its biological plausibility.

In addition to biological hypotheses, the apparent survival difference may be explained by behavioral mechanisms. As there was variability allowed in the trial by permitting clinical decision making, it is possible that observed patient parameters during the trial may have led to different clinician behavior. For example, patients with prior ADT may have responded differently to immediate ADT than other patients, triggering different treatment patterns such as earlier initiation of secondary therapies such as docetaxel. Even without different disease parameters, it is also possible that clinicians treat patients differently in the setting of prior ADT. For example, a clinician may have a lower threshold for declaring ADT a failure if a patient has previously received it, perhaps believing the disease is closer to castration resistance (6). Trial participants in the prior ADT group may thus have received further lines of therapy (eg, docetaxel) earlier than other groups, resulting in superior survival. Unfortunately, there are limited data available from the TOAD trial on secondary treatment regimens beyond ADT receipt, so we cannot assess this hypothesis using existing trial data.

We also considered prior ADT receipt as an indicator of higher risk disease (ie, reflective of selection bias instead of a causal effect on response to immediate vs delayed ADT). We observed more patients with T3-T4 disease and higher PSA at time of diagnosis in the prior ADT group. However, other indicators of disease aggressiveness including Gleason score and PSA at time of trial eligibility were similar between groups. Additionally, more aggressive disease alone would not explain the excellent survival in the prior ADT group randomized to immediate ADT at time of relapse or why this benefit was not seen in the no prior ADT group. Similarly, we thought prior ADT might suggest to clinicians that subsequent ADT could be safely delayed while still adhering to the TOAD protocol. However, time to receipt of ADT in the delayed arm was similar in the prior ADT and no prior ADT groups, suggesting this treatment selection was not significant. We also addressed the fact that prior ADT could simply be an indicator of definitive combination treatment with radiotherapy and found similar results when limiting our analyses only to patients who had undergone radiation therapy. Even if the observed survival benefit is because of more aggressive disease, our findings could still be applied to better select patients who would benefit from immediate vs delayed ADT and allow for sparing harms to a substantial number of patients by avoiding ADT in lower risk groups.

Our primary takeaway is that immediate ADT may be the preferred strategy for patients with PSA-relapsed prostate cancer after local treatment, if they received prior ADT. If our results are supported by further evidence, there are major implications for prostate cancer research and practice.

First, the timing of ADT for recurrent prostate cancer could be modified according to prior receipt of ADT. If a prostate cancer patient has not received ADT prior to recurrence, it may be safe to delay ADT. This stratified approach would allow for improved quality of life in many prostate cancer patients who would never require ADT, such as the 41% of men in the TOAD trial delayed treatment arm who never received ADT. In this way, timing of ADT after biochemical recurrence may be considered analogous to intermittent vs continuous ADT, in that some patients (ie, those without prior ADT) may have equivalent outcomes with delayed ADT compared with immediate ADT (19).

Next, our findings may provide an underlying mechanism for the similar efficacy between intermittent and continuous ADT for advanced prostate cancer treatment. Characteristics predicting benefit from continuous ADT may be similar to characteristics predicting benefit from immediate ADT. Improved targeting of both strategies would allow for avoidance of ADT when there is little to no clinical benefit (ie, allowing for intermittent and/or delayed ADT). Taken one step further, bipolar androgen therapy whereby testosterone is cycled between high and low levels to promote resensitization of castration-resistant cells has shown promising early results representing a paradigm shift in the advanced prostate cancer landscape, including a recent trial where bipolar androgen therapy had similar efficacy to enzalutamide in patients with metastatic prostate cancer progressing on abiraterone (20-22). It is also possible genomic classifiers could aid in identifying patients who may be harmed by ADT and could allow for targeted avoidance of ADT (23).

These implications must be further studied prior to incorporation into treatment plans. Most importantly, future studies and particularly clinical trials need to closely examine the role of prior ADT in patients with recurrent prostate cancer. Whether this characteristic is considered within trial eligibility criteria or for stratification, it should be further studied and adjusted for in analyses. It is also possible there is a biologic difference in recurrent disease conditional on prior receipt of ADT; understanding these differences from a translational science standpoint may allow for the development of new precision diagnostics and androgen cycling to tailor treatment strategies for recurrent disease (21).

Our study findings raise important clinical and scientific questions but must be considered in the context of several limitations. First, we analyzed trial data post hoc, with potential for confounding inherent in retrospective analyses. However, the large observed effect size in our study warrants explanation. Second, analyses are limited to data collected for the TOAD trial. Some data informing our hypotheses, such as receipt of secondary treatments like docetaxel, abiraterone, or enzalutamide, were unavailable and should be explored in future studies. Finally, our study inherits the limitations of the original TOAD trial, including lower than anticipated enrollment, which potentially introduces bias. Still, an explanation for the large observed effect is needed and, even if due to selection bias, would be an important finding to guide ADT use and future study directions.

The TOAD study addressed a critical question for recurrent prostate cancer and found a survival benefit for immediate ADT compared with delayed ADT. We found this benefit was driven primarily by patients who had received prior ADT. Although further research is needed to confirm these results, our findings have important mechanistic, clinical, and research implications.

## Funding

This work was supported by National Cancer Institute at the National Institutes of Health (R01 CA242559 to TAS and AT).

## Notes

**Role of the funder:** The funder had no role in the design of the study; the collection, analysis, and interpretation of the data; the writing of the manuscript; or the decision to submit the manuscript for publication.

**Disclosures:** There are no relevant author conflicts of interest for this study.

**Author contributions:** Conceptualization: KDS, TD, AT, TAS. Data curation: TD, JB. Formal analysis: TD, JB, AT. Funding acquisition: BH, AT, TAS. Investigation: KDS, TD, JB, GMD, AT, TAS. Methodology: TD, AT. Project administration: JS, TAS. Resources: AT, TAS. Software: TD, JB, TAS. Supervision: AT, TAS. Validation: KDS, TD, AT. Visualization: KDS, TD. Writing, original draft: KDS, TAS. Writing, review and editing: All authors.

**Prior** **presentations:** The findings of this study were presented at the American Urological Association Meeting in September 2021.

## Data Availability

The data underlying this article will be shared on reasonable request to the corresponding author.

## References

[1] NCCN Guidelines. *NCCN Clinical Practice Guidelines in Oncology: Prostate Cancer*; 2021. https://www.nccn.org/professionals/physician_gls/pdf/prostate.pdf. Accessed February 5, 2021.

[2] Lowrance W , Breau R, Chou R, et al Advanced prostate cancer: AUA/ASTRO/SUO guidelines; 2020. https://www.auanet.org/guidelines/advanced-prostate-cancer. Accessed February 5, 2021.

[3] Duchesne GM , Woo HH, Bassett JK, et al Timing of androgen-deprivation therapy in patients with prostate cancer with a rising PSA (TROG 03.06 and VCOG PR 01-03 [TOAD]): a randomised, multicentre, non-blinded, phase 3 trial. Lancet Oncol. 2016;17(6):727–737. doi:10.1016/S1470-2045(16)00107-8. 27155740

[4] Fossati N , Gandaglia G, Dell’Oglio P, Montorsi F, Briganti A. Timing of androgen-deprivation therapy for prostate cancer: still a long way to go. Lancet Oncol. 2016;17(8):e313.doi:10.1016/S1470-2045(16)30309-6. 27511149

[5] Leapman MS , Carroll PR. Immediate androgen deprivation: for all or for some? Lancet Oncol. 2016;17(6):683–684. doi:10.1016/S1470-2045(16)00149-2. 27155739

[6] Maitland NJ. Resistance to antiandrogens in prostate cancer: is it inevitable, intrinsic or induced? Cancers. 2021;13(2):327.doi:10.3390/cancers13020327. 33477370 PMC7829888

[7] Duchesne GM , Woo HH, Syme RA, et al A collaborative randomised phase III trial: the timing of intervention with androgen deprivation in prostate cancer patients with a rising PSA (TOAD). TROG 03.06 trial protocol, Version 3; 1 October 2009. https://clinicaltrials.gov/ct2/show/NCT00110162. Accessed April 6, 2022.

[8] Barraclough H , Govindan R. Biostatistics primer: what a clinician ought to know: subgroup analyses. J Thorac Oncol. 2010;5(5):741–746. doi:10.1097/JTO.0b013e3181d9009e. 20421767

[9] McAllister MJ , Underwood MA, Leung HY, Edwards J. A review on the interactions between the tumor microenvironment and androgen receptor signaling in prostate cancer. Transl Res. 2019;206:91–106. doi:10.1016/j.trsl.2018.11.004. 30528321

[10] Niu Y , Guo C, Wen S, et al ADT with antiandrogens in prostate cancer induces adverse effect of increasing resistance, neuroendocrine differentiation and tumor metastasis. Cancer Lett. 2018;439:47–55. doi:10.1016/j.canlet.2018.09.020. 30227222 10.1016/j.canlet.2018.09.020

[11] Boibessot C , Toren P. Sex steroids in the tumor microenvironment and prostate cancer progression. Endocr Relat Cancer. 2018;25(3):R179–R196. doi:10.1530/ERC-17-0493. 29317479 10.1530/ERC-17-0493

[12] Karantanos T , Evans CP, Tombal B, Thompson TC, Montironi R, Isaacs WB. Understanding the mechanisms of androgen deprivation resistance in prostate cancer at the molecular level. Eur Urol. 2015;67(3):470–479. doi:10.1016/j.eururo.2014.09.049. 25306226 PMC5301306

[13] Kumari S , Senapati D, Heemers HV. Rationale for the development of alternative forms of androgen deprivation therapy. Endocr Relat Cancer. 2017;24(8):R275–R295. doi:10.1530/ERC-17-0121. 28566530 PMC5886376

[14] Nair SS , Weil R, Dovey Z, Davis A, Tewari AK. The tumor microenvironment and immunotherapy in prostate and bladder cancer. Urol Clin North Am. 2020;47(4S):e17–e54. doi:10.1016/j.ucl.2020.10.005. 33446323

[15] Ojo D , Lin X, Wong N, Gu Y, Tang D. Prostate cancer stem-like cells contribute to the development of castration-resistant prostate cancer. Cancers. 2015;7(4):2290–2308. doi:10.3390/cancers7040890. 26593949 PMC4695890

[16] Gordon RR , Nelson PS. Cellular senescence and cancer chemotherapy resistance. Drug Resist Updat. 2012;15(1-2):123–131. doi:10.1016/j.drup.2012.01.002. 22365330 PMC3348393

[17] Tsodikov A , Müller WA. Modeling carcinogenesis under a time-changing exposure. Math Biosci. 1998;152(2):179–191. doi:10.1016/S0025-5564(98)10030-5. 9780614

[18] Freedland SJ , Humphreys EB, Mangold LA, et al Risk of prostate cancer-specific mortality following biochemical recurrence after radical prostatectomy. JAMA. 2005;294(4):433–439. doi:10.1001/jama.294.4.433. 16046649

[19] Crook JM , O’Callaghan CJ, Duncan G, et al Intermittent androgen suppression for rising PSA level after radiotherapy. N Engl J Med. 2012;367(10):895–903. doi:10.1056/NEJMoa1201546. 22931259 PMC3521033

[20] Teply BA , Wang H, Luber B, et al Bipolar androgen therapy in men with metastatic castration-resistant prostate cancer after progression on enzalutamide: an open-label, phase 2, multicohort study. Lancet Oncol. 2018;19(1):76–86. doi:10.1016/S1470-2045(17)30906-3. 29248236 PMC5875180

[21] Schweizer MT , Antonarakis ES, Wang H, et al Effect of bipolar androgen therapy for asymptomatic men with castration-resistant prostate cancer: results from a pilot clinical study. Sci Transl Med. 2015;7(269):269ra2.doi:10.1126/scitranslmed.3010563. PMC4507510 25568070

[22] Denmeade SR , Wang H, Agarwal N, et al TRANSFORMER: a randomized phase II study comparing bipolar androgen therapy versus enzalutamide in asymptomatic men with castration-resistant metastatic prostate cancer. J Clin Oncol. 2021;39(12):1371–1382. doi:10.1200/J Clin Oncol.20.02759. 33617303 10.1200/JCO.20.02759 PMC8274807

[23] Feng FY, Huang HC, Spratt DE, et al. Validation of a 22-gene genomic classifier in patients with recurrent prostate cancer: an ancillary study of the NRG/RTOG 9601 randomized clinical trial. *JAMA Oncol*. 2021;7(4):544. 10.1001/jamaoncol.2020.7671 PMC7879385 33570548

